# Healthcare professionals’ experiences and views of providing continence support and advice to people living at home with dementia: “That’s a carer’s job”

**DOI:** 10.1186/s12877-024-04830-8

**Published:** 2024-02-29

**Authors:** Barbara Bradbury, Helen Chester, Miriam Santer, Leanne Morrison, Mandy Fader, Jane Ward, Jill Manthorpe, Catherine Murphy

**Affiliations:** 1 School of Health Sciences, University of Southampton, Southampton General Hospital, Tremona Road, Shirley, SO16 6YD UK; 2 NIHR Policy Research Unit in Health and Social Care Workforce, The Policy Institute, King’s College London, Virginia Woolf Building, 22 Kingsway, London, WC2B 6LE UK; 3 Primary Care Research Centre, School of Primary Care, Population Sciences and Medical Education (PPM), Faculty of Medicine, University of Southampton, Aldermoor Health Centre, Southampton, SO16 5ST UK; 4 Department of Psychology, Faculty of Environmental and Life Sciences, Primary Care Research Centre, Faculty of Medicine, University of Southampton, Highfield Campus, Southampton, SO17 1BJ UK; 5 University of Southampton, B67, West Highfield Campus, University Road, Southampton, SO17 1BJ UK

**Keywords:** Dementia care, Urinary incontinence, Continence management, Primary healthcare professionals

## Abstract

**Background:**

People living with dementia at home and their family carers often feel unsupported by healthcare professionals in managing continence problems. In turn, primary and community-based healthcare professionals have reported lacking specific knowledge on dementia-continence. This study aimed to understand more about healthcare professionals’ experiences and views of supporting people living with dementia experiencing continence problems, as part of developing acceptable resources. Having a nuanced understanding of unmet need would facilitate the design of engaging resources that enable healthcare professionals to provide more effective continence support to people living with dementia at home.

**Methods:**

Semi-structured interviews were conducted with a range of healthcare professionals (*n* = 31) working in primary and community care in the South of England in 2023. Transcribed interviews were uploaded to NVivo 12, then analysed inductively and deductively using a thematic framework.

**Results:**

Continence-related conversations were avoided by many healthcare professionals due to lack of dementia-continence specific knowledge. Many considered that continence problems of people living with dementia were largely outside their remit once a physical cause had been ruled out. This contributed to a lack of priority and proactivity in raising the subject of continence in their consultations. Challenges to providing support included limited consultation time and lack of access to specialist services with availability to support individuals.

**Conclusion:**

There is substantial scope to support primary and community-based healthcare professionals in their provision of continence-related support and advice to people living at home with dementia. This includes addressing knowledge deficits, enhancing confidence and instilling a sense of accomplishment.

**Supplementary Information:**

The online version contains supplementary material available at 10.1186/s12877-024-04830-8.

## Background

Most people living with dementia remain at home and many will experience continence or toilet-use problems as their condition progresses [1–3]. Stigma associated with either a diagnosis of dementia or with continence problems is well documented [4, 5]. The combination of living with both conditions can be highly stressful, both for the person living with dementia and their family carers, and may contribute to social withdrawal, potential isolation and breakdown in care [6–8]. Continence discussions are frequently avoided due to the taboo nature of the subject [9] and the mental and physical stress of care can become too great to handle. Continence and toilet-use problems are frequently the trigger for someone living with dementia to move from their family home into permanent residential care [10, 11].

Family carers (hereafter referred to as carers) have reported difficulty in mentioning continence problems due to embarrassment and stigma associated with the subject. They would appreciate greater support, information and practical advice from healthcare professionals (HCP), particularly their family doctor, primary care and community nurses, about continence and how to manage it [9, 12, 13]. However, these HCPs have acknowledged their limited knowledge around dementia-related incontinence, and their frustration with inadequate resources to provide appropriate support, resulting in avoidance of potentially difficult conversations [12, 14]. The lack of understanding of the nuances of dementia-related continence problems can lead to HCPs offering management strategies that are more appropriate for people without dementia [14]. There is clearly a need to support HCPs to develop a broad knowledge base regarding dementia-continence, and to provide resources that enable them to offer greater support to people living with dementia and their carers on this subject. However, in order to develop resources to support HCPs in this task, as a first stage of intervention development it is necessary to have a greater understanding of their experiences and elicit views on the challenges of providing support [15]. Therefore, in the preliminary stages of a study seeking to address the provision of improved continence-related support specific to people with dementia and their carers, we set out to gain the first in-depth understanding of the current support provided by a wide range of primary care (practitioners based in general practices) and community-based (practitioners employed by NHS Community Trusts) HCPs.

### Aim

To understand primary care and community-based HCPs’ experiences and views on providing continence-related support to people living at home with dementia and their carers.

## Methods

### Design

A qualitative study design of semi-structured interviews with HCPs practicing in the South of England. Ethical approval was obtained from the University of Southampton (ERGO 75111) and the Health Research Authority (IRAS 318255). The Consolidated Criteria for Reporting Qualitative (COREQ) Research guideline statement assisted reporting [16]. A checklist is provided in the supplementary materials (see Additional file 1).

### Participant sampling and recruitment

A purposive sample of HCPs working in community and primary care, with experience of providing continence or toilet-use related advice or support to people living at home with dementia and their carers ensured a range of primary and community healthcare professionals were included [17]. Research and Development (R&D) departments in Community Trusts in London and the South of England were contacted via email by BB and CM to raise awareness and gain interest in the study. The National Institute for Health and Care Research (NIHR) Applied Research Collaboration (ARC) Wessex also assisted with recruitment of primary care practitioners. Organisational R&D leads subsequently introduced potential participants to the research team, who were contacted by BB via email and sent a Participant Information Sheet (PIS) and copy of the Consent Form in order to make an informed decision to participate. The PIS included the aims of the study, method of data collection and storage of data. It also underlined that participation was purely voluntary, anonymity was guaranteed and there was the right to withdraw, without prejudice, at any stage of their involvement. Participants (*n* = 31) were recruited from NHS general practices (*n* = 10) and Community Trusts (*n* = 3). They included nurses from a variety of disciplines, general medical practitioners (GPs) and allied health professionals. No participant was known previously to the research team members. There was no limit imposed on the number of participants in any specific role or discipline. Thus, all HCPs who expressed an interest in the study were considered for inclusion, provided they met the criteria of having given continence or toilet-use related advice or support to people living at home with dementia and their carers. Participant characteristics are reported in Table 1.

**Table 1:** Participant characteristics (*n* = 31)

Professional Role	(Identifier)	n	Professional Role	(Identifier)	n
Care Home Co-ordinator	CHC	1	Advanced Nurse Practitioner	ANP	1
Community Paramedic	CP	1	Assistant Nurse Practitioner	Asst.NP	1
General Practitioner	GP	6	Community Matron	CM	1
Occupational Therapist	OT	3	Community/District Nurse	CN/DN	7
Physiotherapist	Phy	1	Practice Nurse	PN	2
Social Prescriber	SP	1	Specialist Nurse Practitioner (Continence/dementia/frailty)	SNP	6
**Time in current role (Years)** Mean 11.5 (Min 2; Max 30)			**Sex** Female: *n* = 28; Male: *n* = 3		

### Data collection

Audio recorded telephone interviews of 30–40 min were conducted between November 2022 and February 2023 by BB. All participants gave verbal consent, which was recorded on a separate audio file prior to the start of their interview. This avoided potential delays due to paper copies being mislaid in the post, or people having difficulty with producing and returning email signatures on electronic copies of the consent form. The topic guide (Table 2) was informed by previous research [12] and designed to address the aims of the study.

**Table 2:** Topic guide summary

1. Please tell me about your current role and previous work experience
2. What experience do you have of delivering continence-related support to people who are living with dementia, their family carers/friends or homecare workers?
3. How do you feel about delivering this type of support?
4. How central is it to your work? How often do you provide this type of support?
5. Who usually initiates the conversation?
6. How confident are you in having these conversations?
7. How have you learnt about continence care for people living with dementia?

Participating NHS organisations signed a non-commercial Participant Identification Centre (PIC) Agreement. Five general practices, involving eight participants, elected to be reimbursed for their time as per the financial schedule of the PIC Agreement. The remaining organisations opted for their participants to receive a gift voucher of £25 each in acknowledgment of their contribution; all interviewed were given a certificate of participation.

### Data analysis

Interview data were analysed thematically using the Framework Method [18], following the process described by Gale et al*.* [19]. This method is used extensively for thematic analysis of semi-structured interviews in health research [20–22]. It is particularly suitable for analysing qualitative data covering similar issues by enabling systematic organisation of the data into themes, through comparison within and between participant cases [19].

Interviews were transcribed verbatim and identifying data removed. Familiarisation with the data occurred during and after transcription, prior to initial coding. Transcriptions were uploaded to NVivo 12 and BB completed the initial coding of all transcripts. Both inductive and deductive approaches [23] were used concurrently to identify all salient themes in the data. Deduction was used to identify codes that addressed pre-defined areas of interest to the project (for example, current level of HCP continence support and their prior knowledge of the related dementia-continence subject matter). Inductive coding ensured identification of additional codes and subsequent sub-themes (such as relevance to role and caseload volume).

The first few coded transcripts were discussed with members of the research team (CM, HC) and codes refined. The remaining interview transcripts were coded using the coding frame and new codes discussed with the team and added as they were identified from the data. During iterations of coding, transcripts were checked to ensure consistency was maintained across the dataset. Codes were grouped into sub-themes of related concepts and overarching themes subsequently identified by members of the research team (BB, CM, HC). An example of charting data into the framework matrix is provided in the supplementary materials (see Additional file 2).

## Results

Thematic analysis of the interview data identified seven sub-themes and two overarching themes: clinician factors (Theme I) and system factors (Theme II) that affected the initiation of continence conversations with people living with dementia and their carers. The system in the context of this research refers to various elements and organisation of the health service that impact the provision of care by primary and community healthcare professionals. The sub-themes are summarised below with illustrative quotes: words in round brackets *(words)* contextualise the quotation and omission of verbal text between sentences is indicated thus *[…]*.

## Theme I: Clinician factors

**Fig. 1:**
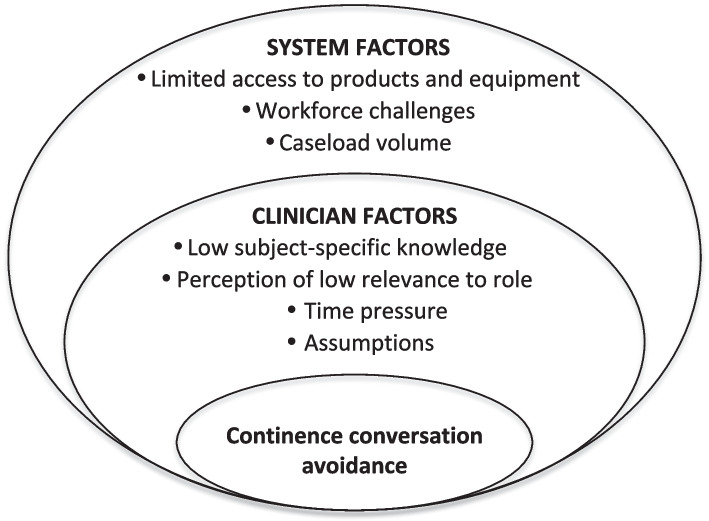
THEMES and Sub-themes

Experiences of raising the subject of continence with people living with dementia and their carers varied according to profession. It was acknowledged by GPs that continence is *“a big problem that I don’t think we address very well”* (HP 4: GP). Several reasons were given for this, summarised through the four sub-themes of low subject-specific knowledge, perception of low relevance to their role, time pressure and the assumptions made by HCPs (Fig. 1).

Excepting the various dementia specialist practitioners in this subject area, participants reported limited understanding of the nuances of continence for people living with dementia: most recalled no specific educational input on continence and dementia. This affected the confidence they had in discussing the subject and provision of continence management guidance:

*“A lot of people would say, ‘why are they getting incontinent and why is this happening?’ I didn’t feel confident in that kind of area. […] Actual continence knowledge, I wouldn’t say I was that confident”* (HP 22: CN).

A lack of acknowledgment of the nuances of dementia-related continence problems was evident in the following comment, in which the focus of the study was questioned:

*“It’s interesting because the study is just about dementia patients but I think the whole, I think it’s (continence is) a huge problem for everyone. I don’t think dementia is the only … dementia is a small part of where the problem lies and I don’t think my knowledge is very good at all, if I’m honest. […] I think the dementia side is somewhat irrelevant. I think it’s any patient or any carers. I think the dementia, yes it adds an extra level of complexity, but I think the service for anyone with incontinence of any description is poor”* (HP 2: GP).

A second sub-theme regarded the perceived relevance to their role of holding continence-related conversations. There was widespread contention among GPs, primary care and some community nurses that their remit was to diagnose or exclude a medically treatable cause (e.g. a urinary tract infection or faecal impaction). Advising people living with dementia on how to manage continence was viewed as the province of other HCP specialists. Homecare workers (not professionally qualified) were also reported to be highly involved in continence management and frequently deemed to be the professional group more appropriate to discuss continence with people who have dementia and their carers.

Various HCPs referred to themselves as a ‘Jack of all trades’, whose role in this was to signpost people living with dementia to continence specialist practitioners or homecare workers for continence support. Some HCP roles, such as that of Social Prescriber, were employed to signpost patients to an appropriate service or professional:

*“So we can help in that way of people who are struggling to understand why they’ve become incontinent; we can help them access the service that will be able to support them with that conversation. So we’re a kind of link to find services that will help them with that”* (HP 5: SP).

Many generalist HCPs agreed that continence specialist practitioners are best placed to have conversations about continence management with people living with dementia. However, some of these specialist practitioners or therapists thought that the subject should be regarded as a priority amongst all community and primary care HCPs, as observed by a participant:

*“I think continence needs to be brought way further forward in the list of priorities and importance for everyone who is caring for patients. So, not just the community nurses but every single person, any of the therapists, any doctors, everybody should be seeing continence as an important issue that we should be addressing”* (HP 30: Phy).

With respect to time pressure, limited time during an appointment was frequently reported by GPs and other HCPs to be a contributing factor:

*“I wouldn’t directly ask in my consultation unless someone brings it to the consultation. […] *I simply haven't got time to tackle everything in every appointment" (HP2: GP).

Time was also acknowledged to be a system challenge, impacted by caseload and the time formally allocated to each consultation or patient visit, which is discussed below.

A fourth sub-theme related to HCPs’ assumptions about the willingness of people with dementia and their carers to engage in a continence-related conversation, reporting that: *“People are obviously embarrassed to speak about it and they don’t want to admit that there is a problem”* (HP 22: CN). Some HCPs spoke of the negative reaction they sometimes received from people living with dementia if they tried to raise the subject, which caused anxiety among some HCPs:

*“If we have someone come on the books that they say they have dementia and they are incontinent, it sets alarm bells in one way with us because we know that it might be a bit of a battle”* (HP 12: SNP).

## Theme II: System factors

Three main system factors impacted HCPs’ proactive support: limited access to products and equipment, workforce challenges and caseload volume. With respect to products and equipment, knowledge of the system, what was available for people with dementia and how things might be accessed affected the advice that HCPs offered. For some, this resulted in their choosing to avoid certain subjects, most notably around the subject of continence products. The opposite was also evident, whereby some HCPs used their knowledge to benefit people living with dementia. For example, a community nurse reported augmenting a continence assessment to increase the likelihood of it being accepted by the specialist continence service, stating:

*“We do exaggerate the bladder diaries and we put as much clinical information into the assessments as we possibly can because if you don’t, you know that they (Specialist Continence Service) won’t give you what you want (continence products). They almost treat every patient the same, they don’t take into consideration all the other factors, especially patients with dementia*” (HP 7: Asst.NP).

Limited opportunity to provide continuity of care was identified as a workforce challenge which impacted on the confidence and ability of some HCPs to give continence support to people living with dementia. Practitioners remarked on the importance of building a relationship with the person living with dementia, enabling them to feel more comfortable and confident in their care and support. They also felt this made it easier for the person living with dementia to ask for their help. Recognising that dementia affects people in various ways, knowing their patients enabled HCPs to better support them individually. Most acknowledged that it took time to understand how each person living with dementia was affected by it and underlined the importance of building a relationship with them in order to address their specific care needs.

Another workforce challenge identified was a lack of confidence in the system to provide additional support. This was given as a rationale to avoid initiating continence-related conversations:

*“I don’t actively seek out if a patient has continence problems particularly, because I think the (continence) service is not particularly good, if I’m honest”* (HP 3: GP).

Conversely, some reported that good access to continence specialists in the community enhances levels of continence support:

*“You need somebody, it’s about having continence leads in the community, their continence service. If they (community nurses) don’t feel supported and comfortable to talk about it (continence) they need to link in with the continence service from their team, who should provide training for their team and work out, have discussions at a local level, about breaking down those barriers”* (HP 25: CM).

Staff shortages had contributed to the caseload volume of HCPs and its resultant system challenge identified in this sub-theme. The pressures on community nursing services and declining numbers of community nursing staff have impacted their priorities. Linked to the relatively low numbers of practitioners was their shortage of time in which to converse with their patients. A consequence of this was that continence issues had become a low priority for many HCPs:

*“If a community nurse is going in to see a patient, they’re completely rushed off their feet so when they go in to do a patient … say they’ve gone in to do an insulin injection, they’re not going to be interested whether or not the patient has been incontinent. That’s a carer’s (paid homecare worker’s) job. So they’ll go in and do the diabetic injection: ‘See you Mrs Thingy, well done, your blood sugar is great this morning’. But the continence gets left”* (HP13: DN).

Thus, several factors have reportedly impacted the opportunity for, or priority of, HCPs to provide adequate support to people living with dementia at home and their family carers on the subject of continence.

## Discussion

This paper provides the first in-depth analysis of a wide range of primary and community care HCPs’ views and experiences on providing continence-related support to people living at home with dementia. Views on involvement in the provision of support were influenced by four main factors: HCPs’ specialist knowledge; workforce and caseload considerations, giving rise to the low priority they gave to the subject; and perceived relevance of their involvement.

Carers of people living with dementia and continence-related problems expect primary care professionals to be a source of advice and support [12, 13]. However, we found that a variety of healthcare professionals, including GPs, community paramedics, primary care and community nurses, do not think it is feasible to take this on as part of their role beyond referring people for possible treatment of an underlying medical cause for the continence problem, or providing such a consultation. They also report referring people to specialist continence services, primarily for them to access NHS-prescribed, free of charge, absorbable continence products. Practical, and presumably emotional, help with dealing with incontinence episodes was seen to be the province of non-qualified social care practitioners.

Several HCP groups, including GPs, community nurses and paramedics described themselves as generalists, lacking detailed knowledge of dementia-related continence. This reportedly affected their willingness to proactively initiate a conversation on continence with a person living with dementia or their carer. Notably, GPs admitted avoiding such conversations even when undertaking an annual dementia review. Iliffe et al*.* [24] similarly noted substantial knowledge deficits and poor support given by primary care HCPs to people living with dementia and their carers. They called for greater proactivity amongst HCPs to increase their knowledge in this subject area and raise the subject during consultations, in order to provide information and advice (*ibid*). There does not seem to have been progress on such recommendations.

The value of having an existing relationship with their patients was acknowledged by HCPs to ease the introduction of a potentially difficult conversation regarding incontinence with people living with dementia and their carers, although not all would necessarily be in roles enabling this to develop. The positive impact on the therapeutic relationship facilitated by personal continuity of care is well documented in the general healthcare [25, 26] and mental health literature [27, 28]. The consistent delivery of care to a patient by a primary care HCP enables the building of a relationship and facilitates observation of changes in a person’s health over time [26]. However, providing personal continuity of care is becoming more challenging within primary care in the NHS [29, 30]. It is worth exploring further as to whether this contributes to the challenge of giving proactive continence related advice and support to people living with dementia, although having a named GP (as required for all patients aged over 75 years in England) does not appear to make a substantial difference [31].

Pressure of time was given as a reason for avoidance of continence-related conversations. Community nurses and GPs focussed on other aspects of care and assessment. In keeping with earlier research [24], continence was not a subject prioritised for discussion within the limited time available. Several reasons may contribute to this. For example, HCPs spoke of their frustration of problems within the system in which they worked and felt they had little to offer patients should they expose a need. Drennan et al*.* ([32], p.340) refer to “therapeutic nihilism” (i.e. an attitude that nothing can be done to prevent incontinence), which may also contribute to a lack of engagement by HCPs. However, it is possible to help people with dementia effectively manage potential incontinent episodes [33]. Also, not everyone living with dementia experiences continence problems [7]. It is important for HCPs to be mindful of how their perceptions impact on their behaviour [34].

On the other hand, in common with other research [9, 35], some HCPs perceived reticence among people with dementia and their carers to seek help and mention continence. The stigma associated with a diagnosis of dementia is compounded by the taboo nature of incontinence [9, 36], leading some people to avoid discussing the subject with a HCP in order to preserve their dignity and hide a sense of shame or embarrassment [13, 37]. Some people with dementia and their carers may normalise the situation as part of ageing and dismiss the idea that they have a problem [38, 39]. However, a common reason that people living with dementia move into a care home is the difficulty facing carers to deal, largely alone, with incontinence [10, 11, 13]. This suggests that it is in the best interests of people with dementia and their carers to be given advice and help in managing continence before reaching crisis point. A more proactive approach from HCPs to continence-related concerns could help avoid such crises, and giving management advice and support could usefully be seen as part of the role of all HCPs who consult with people living with dementia [3, 40]. However, HCPs need better support to overcome the challenges of achieving this goal and this may need to be more explicitly addressed in professional qualifying programmes and employment-based learning. For the immediate future, the new findings from our work will inform the development of a bespoke intervention that aims to enhance the knowledge of HCPs in dementia-continence, enabling them to give more effective support and advice in continence management and help to prolong the independence of people living with dementia.

### Study limitations

There are several limitations to this study, which relate to aspects of sampling. Despite considerable effort, we were unable to engage with substantial numbers of Practice Nurses. Therefore, their views as to whether they perceive this area of clinical practice to be outside their remit are largely unexplored. Our sample (*n* = 31) comprised seven professional groups, including nurses with varying roles and from six clinical specialisms. This breadth of HCPs makes it difficult to generalise findings within professional groups. However, the aim of the research was to gather wide-ranging views across the participant cohort and has been successful in that regard. Also, the sample was predominantly white British (*n* = 22), female and based in the South of England, which was representative of its population but limits wider transferability of the findings. Finally, the role of medication in the treatment of dementia was not explored and might be considered to be a limitation of the study, as medication to treat dementia symptoms can cause incontinence. However, the specific cause of incontinence in people with dementia was not the focus of the study, and therefore medication was not included.

## Conclusion

This study has provided a nuanced understanding of the challenges faced by primary care and community-based HCPs about providing continence advice and assistance to people living at home with dementia and their carers. Despite the well-documented need for better continence advice and support for people living with dementia and carers, HCPs may consider this subject to be largely outside their remit. Any intervention aiming to promote continence-related discussion must overcome this hurdle by facilitating greater understanding of the subject matter, enhancing confidence and instilling a sense of accomplishment when support is provided.

### Supplementary Information

**Additional file 1.** Consolidated criteria for reporting qualitative research checklist.

**Additional file 2.** Charting data into the framework matrix.

## Data Availability

Data collected and analysed during this study are available from the corresponding author on reasonable request.

## Abbreviations

GP: General Medical Practitioner
HCP: Healthcare Professional
NHS: National Health Service
