# Supplementary material for: Healthcare professionals’ experiences and views of providing continence support and advice to people living at home with dementia: “That’s a carer’s job”
Source: BMC Geriatr. 2024 Feb 29;24:213. doi: 10.1186/s12877-024-04830-8 (PMC10905774; doi:10.1186/s12877-024-04830-8)
Supplement: Supplementary file 2 — Additional file 2. Charting data into the framework matrix. [file 12877_2024_4830_MOESM2_ESM.pdf]

Additional file 2: Charting data into the framework matrix

Theme I: Clinician Factors

Subject-specific knowledge	Time constraints	Assumptions	Relevance to role
They don't often put them (dementia and continence) together. […] I think it needs to be a really personal, how to approach, how to look at a patient and put it altogether with the dementia. (HP13: DN)	If there's a list and if you have someone, […] if there is syringe drivers for end of life patients, or essential medication, wound care, a continence assessment will get left to the next day because it's not seen as essential and that can go on for weeks. (HP23: SNP)	Someone supporting someone with dementia, who is their family carer, they might actually not want to admit that they are struggling because they want to do the best they can for their family member. (HP6: CHC)	Cleanliness, pad changing, pressure area care is all down now to the carers (paid homecare workers). […] When it is discussed we sweep it under the carpet and then think about referring to the continence nurse and getting incontinence pads. (HP19: OT)
A lot of people would say, 'why are they getting incontinent and why is this happening'? I didn't feel confident in that kind of area. […] Actual continence knowledge, I wouldn't say I was that confident. (HP22: CN)	District nursing has become a bit of a task orientated type of role where you go in and do what you need to do and you leave and you don't really go digging or asking questions […] I don't think it's always been like that but that's what it seems to be turning into now given time pressures (HP20: CNS)	When they've got mild or moderate continence and the spouse, or the (family) carer, is fairly on top of things and confident, then I may not ask about the continence on the basis that they may bring it up first. So, they are much better placed to mention the issues at that day-to-day consultation. (HP1: GP)	Once we have an established problem and there's no physical solution to the problem, then we enter into what I would deem a nursing world rather than a medical world and therefore my teaching … I don't remember ever having teaching about how to advise about continence, and it's not something that I see I should advise on. (HP2: GP)
I don't think (I've had) anything (formal educational input) that would be just specifically with those two (dementia and continence issues) in mind together. (HP3: GP)	I think community nurses today are technicians rather than nursing care. Not that they don't care but they have 10 minutes to do something and they are very focused on that task. I don't think it's holistic anymore. I think that's probably a time constraint. (HP26: ANP)	If we have someone come on the books that they say they have dementia and they are incontinent, it sets alarm bells in one way with us because we know that it might be a bit of a battle. (HP12: SNP)	When this came up in the Practice Nurse forum, there was quite a few comments of: 'Well, that's not part of our role'. […] Not all general practice nurses actually want to pick this (continence) up and follow it up. (HP8: PN)
I think there's a huge gap in knowledge.[…] It's really about education. But it's time and I don't really feel that enough time is given to continence. (HP23: SNP)	When we're rattling through things we don't proactively ask people if they need help with the continence when we are doing a bit of a dementia review. (HP14: GP)	I think a lot of people with dementia feel very inadequate and then going on about their continence is just about as mean as it can be really. (HP13: DN)	I think it's out of our remit. […] We're doing acute visiting. I think for us, referrals like highlighting the problem and referring on is more of what we do rather than informing patients with detail. (HP10: CP)

Theme II: System Factors

Access to products and equipment	Workforce challenges	Caseload volume
When it comes to catheterisation it's much harder now for the GP – we don't have the catheterisation kit that we used to have. (HP1: GP)	I don't actively seek out if a patient has continence particularly, because I think the service is not particularly good, if I'm honest. (HP2: GP)	The trouble is the district nurses are now so very pushed with so many other things that it's difficult for them. (HP9: SNP)
The pad team are often refusing items and we think, well this person has got dementia and they don't really know what they are doing. They might use more pads than they (continence team) would allow. (HP11: CN)	District nursing has become a bit of a task orientated type of role where you go in and do what you need to do and you leave and you don't really go digging or asking questions, which is a shame because I don't think it's always been like that. (HP20: SNP)	We only have 10 minutes with a patient, so if we were to ask every conceivable symptom that might be associated with dementia individually we just wouldn't have time. (HP3: GP)
Once you've decided there's a problem, you are a bit limited to what you can really do. (HP4: GP)	I think the district nurses and community nurses are so busy that continence always gets put to the back and it needs to be put to the front. […] But we don't have capacity because we don't have enough staffing in our services. (HP30: Phy)	I've worked in (three different London Boroughs) … and they all have issues with staffing and just too many patients to see and not enough time in the day. (HP20: SNP)
You prescribe pads, or you ask the service to prescribe pads because we can't actually do that […] and we have to operate within quite strict parameters otherwise the pads won't be authorised, and then the pad service authorise or not, what they suggest. (HP16: DN)	You need somebody, it's about having continence leads in the community, their continence service. If they (community nurses) don't feel supported and comfortable to talk about it (continence) they need to link in with the continence service from their team, who should provide training for their team and work out, have discussions at a local level, about breaking down those barriers. (HP25: CM)	If a community nurse is going in to see a patient, they're completely rushed off their feet so when they go in to do a patient, say they've gone in to do an insulin injection, they're not going to be interested whether or not the patient has been incontinent. That's a (paid) carer's job. So they'll go in and do the diabetic injection: 'See you Mrs Thingy, well done, your blood sugar is great this morning'. But the continence gets left. (HP13: DN)
We're not allowed to prescribe continence products without them seeing our continence nurse […] and also, we're constrained by the formulary even once they hit the continence nurse. The catheters and stuff we won't prescribe. (HP2: GP)	I think the main issue is: What's available locally? Their frustrations are, how do I get through the system quicker? We've got a continence team but actually, it took a while for them to come in and see a patient. (HP1: GP)	It's really time and staff capacity. They are so incredibly stretched the district nurses. […] I don't think continence is given the priority it should be. (HP23: SNP)
